# A multiplex reverse transcription-nested polymerase chain reaction for detection and differentiation of wild-type and vaccine strains of canine distemper virus

**DOI:** 10.1186/1743-422X-7-86

**Published:** 2010-05-01

**Authors:** Wei Si, Shun Zhou, Zhao Wang, Shang-jin Cui

**Affiliations:** 1 State Key Laboratory of Veterinary Biotechnology, Harbin Veterinary Research Institute, Chinese Academy of Agricultural Sciences, Harbin, China; 2 Qingdao Agricultural University, Qingdao 266109, China

## Abstract

A multiplex reverse transcription-nested polymerase chain reaction (RT-nPCR) method was developed for the detection and differentiation of wild-type and vaccine strains of canine distemper virus (CDV).  A pair of primers (P1 and P4) specific for CDV corresponding to the highly conserved region of the CDV genome were used as a common primer pair in the first-round PCR of the nested PCR. Primers P2 specific for CDV wild-type strains, were used as the forward primer together with the common reverse primer P4 in the second round of nested PCR. Primers P3, P5 specific for CDV wild-type strain or vaccine strain, were used as the forward primer together with the common reverse primer P4+P6 in the second round of nested PCR. A fragment of 177 bp was amplified from vaccine strain genomic RNA, and a fragment of 247 bp from wild-type strain genomic RNA in the RT-nPCR, and two fragments of 247 bp and 177 bp were amplified from the mixed samples of vaccine and wild-type strains. No amplification was achieved for uninfected cells, or cells infected with Newcastle disease virus (NDV), canine parvovirus (CPV), canine coronavirus (CCV), rabies virus (RV), or canine adenovirus (CAV). The RT-nPCR method was used to detect 30 field samples suspected of canine distemper from Heilongjiang and Jilin Provinces, and 51 samples in Shandong province. As a result of 30 samples, were found to be wild-type-like, and 5 to be vaccine-strain-like. The RT-nPCR method can be used to effectively detect and differentiate wild-type CDV-infected dogs from dogs vaccinated with CDV vaccine, and thus can be used in clinical detection and epidemiological surveillance.

## Introduction

Canine distemper (CD) is a highly contagious and fatal disease of dogs caused by the canine distemper virus (CDV), which is a single-stranded negative RNA virus belonging to the *Morbillivirus *genus within the *Paramyxoviridae *family. Other members of the genus include measles virus (MV) and rinderpest virus (RPV). The genome of CDV is approximately 15,690 nucleotides (nt) in length, containing several genes encoding N, P, M, F, H, and L proteins. Only one serotype has been characterized [1].

A large number of dogs, minks, foxes die from CDV infections every year, causing significant economic losses[2]. Previous studies[3,4] have reported that vaccinated dogs were infected with CDV in Europe and Japan. Harder et al. also reported that there are marked differences between wild-type and vaccine strains of CDV[5], thus whether CDV vaccine strains are able to provide protection from the current strains of CDV remains a question. It is difficult and necessary to discriminate between wild-type and vaccine strains because the attenuated CDV vaccine is used widely in China. So a method to specifically detect the wild-type CDV strains is necessary. The multiplex reverse transcription-nested polymerase chain reaction (RT-nPCR) method could be used to effectively detect and differentiate between wild-type CDV-infected dogs from dogs which were vaccinated with CDV vaccine, which would make it useful in clinical diagnosis and epidemiological monitoring.

## Study Design

With the help of Oligo6 software, six primers were designed based on the genomic sequences of CDV strains published in GenBank (CDV strains: Onderstepoort, Convac, A75/17, Rorkborn, Snyder Hill, Lederle, et al). Forward primers P1 (5'-AAATCCTGTGTTACCCGCTC-3'), P2 (5'-TGGTGGCTCTGCAATATGAA-3'), and P3 (5'-AATGAATGGATGCCTGGGGTTT-3') were used as the primer specific for CDV species, wild-type strains, and vaccine strain Onderstepoort, respectively. Primer P4 (5'-ACGTCCTGGACCCTAAGTTTTG-3') was used as a shared reverse primer. P5(5'-GGTTTTATAAAAGATT), p6(5'-ATCTAGAGGTAA-3') were used as the primer specific for different CDV vaccine strains. Primer pairs P1/P4, P2/P4, P3/P4 and P5/P6 were expected to generate a product of 600, 247, 177 bp and 177 bp, respectively. CDV vaccine (CDV-A strain) and wild-type strains are maintained in the Harbin Veterinary Research Institute. A total of 30 field samples (lung, spleen, liver, or bladder) were collected from different farms in Heilongjiang and Jilin provinces of China.

### Multiplex reverse transcription-nested polymerase chain reaction

Total RNA was extracted from the infected cells with TRIzol^® ^reagent in accordance with the manufacturer's instructions. cDNA synthesis reaction was performed by polymerase chain reaction as described elsewhere using primer Moloney murine leukemia virus reverse transcriptase (M-MLV RT)[6-10]. The first-round PCR was performed with primers P1 and P4. A nested PCR was performed in a total volume of 25 μL containing the first-round PCR products diluted tenfold as well as each of primers P2/P4, P3/P4, or P2, P3/P4[10-12]. The RT-nPCR products were visualized by electrophoresis in a 2% (w/v) agarose gel.

### Specificity, sensitivity, and repeatability tests

RT-nPCR was used to detect the cells infected with CDV vaccine strain, wild-type strain, mixed CDV vaccine and wild-type strains, CPV, CAV, CCV, RV, NDV, and uninfected cells to test its specificity. Extracted RNA from serially diluted (10^4^, 10^3^, 10^2^, 10^1^, 10^0^, 10^-1^, 10^-2^, 10^-3 ^TCID_50_) CDV cell cultures (10^6.5 ^TCID_50_/mL) were assayed by RT-nPCR to determine its sensitivity. RT-nPCR was performed to identify cells infected with CDV vaccine strain, wild-type strain, mixed CDV vaccine and wild-type strains, field samples from Heilongjiang and Jilin province, CPV, CAV, CCV, RV, NDV, and uninfected cells three times to validate the repeatability of the test.

30 samples in Heilongjiang and Jilin provinces, and Fifty-one field samples from dogs, raccoons, foxes, and minks in Shandong provinces were assayed by RT-nPCR; the background of the 51 field samples is listed in table 1. All the positive field samples wild-type strain were confirmed by Rapid test which BioNote, Inc. produced.

**Table 1:** Age, sex, vaccination record, clinical form of distemper and diagnosis for dogs with naturally occurring distemper^a^

No	origin	sex	age	Vaccination record	Clinical Form of distemper	RT-nPCR
1	dog	M	5	vacc	C	V-/W+
2	dog	M	3	vacc	C	V+/W+
3	dog	M	3	vacc	C	V-/W+
4	dog	F	2	vacc	C	V+/W-
5	dog	F	36	no	N	V-/W+
6	dog	M	6	vacc	C	V-/W-
7	dog	F	6	vacc	S	V+/W-
8	dog	M	6	vacc	S	V-/W+
9	dog	F	5	vacc	C	V-/W-
10	dog	F	4	vacc	C	V+/W+
11	dog	M	8	vacc	C	V+/W-
12	dog	M	6	vacc	C	V-/W-
13	dog	F	6	vacc	C	V+/W-
14	dog	F	26	NI	N	V+/W-
15	dog	F	5	vacc	S	V+/W-
16	dog	M	3	vacc	C	V-/W-
17	dog	M	3	vacc	C	V-/W-
18	dog	M	9	NI	S	V+/W-
19	dog	M	4	NI	N	V-/W-
20	dog	F	4	no	N	V-/W+
21	dog	F	3	no	S	V-/W+
22	dog	F	3	no	C	V-/W-
23	dog	F	6	no	C	V-/W-
24	dog	F	6	no	C	V+/W-
25	dog	M	2	no	C	V-/W+
26	dog	M	2	no	S	V-/W-
27	dog	F	2	no	S	V+/W-
28	dog	M	3	no	S	V-/W-
29	dog	M	4	no	S	V-/W-
30	dog	M	3	no	S	V-/W+
31	dog	F	4	no	C	V-/W-
32	raccoon	F	5	vacc	C	V+/W+
33	raccoon	F	3	vacc	C	V-/W-
34	raccoon	F	3	vacc	C	V-/W+
35	raccoon	F	5	vacc	C	V-/W+
36	raccoon	M	5	vacc	S	V-/W-
37	raccoon	M	5	vacc	S	V-/W+
38	raccoon	M	5	vacc	C	V-/W-
39	raccoon	F	4	vacc	C	V+/W-
40	raccoon	F	4	vacc	C	V-/W+
41	raccoon	F	4	vacc	C	V-/W-
42	fox	F	3	vacc	C	V-/W-
43	fox	M	3	vacc	C	V-/W+
44	fox	M	5	vacc	C	V+/W+
45	fox	M	6	vacc	C	V-/W+
46	fox	M	6	vacc	C	V-/W+
47	mink	F	3	vacc	S	V-/W-
48	mink	F	4	vacc	C	V+/W-
49	mink	F	5	vacc	C	V-/W+
50	mink	M	5	vacc	C	V-/W-

51	mink	M	3	vacc	C	V+/W-

^a^Abbreviations: F, female; M, male; C, catarrhalic; S, systemic; N, nervous; V+, vaccine strain positive; W+ wild type positive; V-, vaccine strain negative; W-, wild type negative

### Phylogenetic analysis and detection of CDV in field samples by RT-nPCR

Two of the field samples from Heilongjiang province were selected for amplification of the H gene of CDV by RT-PCR with primers P5 (5'-CCAATTCATCCAAGCTGTCC-3') and P6 (5'-GGGATTTGAACGGTTACATGAG-3'). The amplified products were cloned and sequenced, and the sequences were aligned with the H genes of a number of CDV strains available in GenBank using the MegAlign function of the DNAStar software package. Thirty field samples from dogs, foxes, and raccoons in Heilongjiang and Jilin provinces were also assayed by RT-nPCR.

## Results

### Determination of the application conditions of the multiplex RT-nPCR

After the application of the first-round PCR, primers P2 and P4, and P3, P4, P5 and P6 together, were used to amplify the vaccine and wild-type strains, respectively, at different anneal temperatures. According to the result, when the anneal temperature was from 49-54°C, there was only one specific band. Primers P2, P3, P4, P5 and P6 were used to perform RT-nPCR with different anneal temperatures. Only one specific band was observed at an anneal temperature from 49.5-54.5°C, with the most distinct band appearing at 51.5°C. Thus, 51.5°C was chosen for the RT-nPCR.

### Specificity of the multiplex RT-nPCR

**Figure 1:**
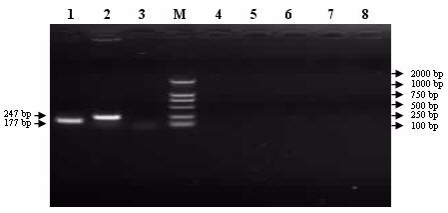
**Amplification of genomes of different easily infected canine viruses by multiplex reverse transcription-nested polymerase chain reaction**. Lane 1: positive control of canine distemper virus (CDV) vaccine strain; lane 2: positive control of CDV wild-type strain; lane 3: uninfected cells control; lane M: DL2000 DNA Marker; lane 4: canine parvovirus; lane 5: canine adenovirus; lane 6: canine coronavirus; lane 7: rabies virus; lane 8: Newcastle disease virus.

A fragment of 247 and 177 bp was amplified from CDV wild-type strain and the vaccine strain, respectively. Two bands of 247 and 177 bp were detected simultaneously from the mixed genomic RNA of the CDV wild-type and vaccine strains. Amplification was not possible for non-CDV viruses, such as CPV, CAV, CCV, RV, NDV-infected cells, and uninfected cells by RT-nPCR (Fig. 1).

### Sensitivity, applicability, and repeatability of the multiplex RT-nPCR

Serial 10-fold dilutions of CDV vaccine strain were subjected to amplification by multiplex RT-nPCR. The lowest limit of detection with this method was shown to be 0.1 TCID_50_. Of the 30 field samples, 20 tested positive for CDV, among which, 15 showed presence of wild-type viruses, and 5 showed presence of vaccine strain. Three independent inter- and intra-assay replicates of the multiplex RT-nPCR gave consistent results, indicating the repeatability of the assay.

### Phylogenetic analysis based on H gene

**Figure 2:**
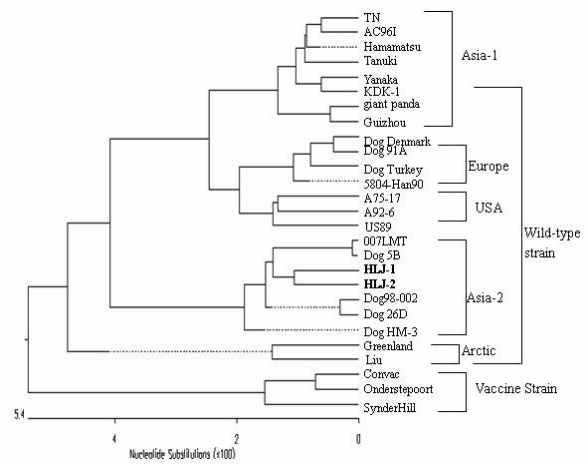
**Phylogenetic tree based on H gene sequences**. GenBank accession numbers of the strains quoted are as follows: AC96I (AB212963), TN (AY390347), Hamamatsu (D85754), Tanuki (AB016776), Yanaka (D85755), KDK-1 (AB025271), giant panda (AF178038), Guizhou (AY359613), US89 (Z47764), A92-6 (Z54166), A75-17 (AF164967), Dog Denmark (Z47761), Dog 91A (AF478544), Dog Turkey (AY093674), 5804-Han90 (X85000), 007LMT(AB212729), Dog5B (AY297453), Dog26D (AB040766), Dog98-002 (AB025270), DogHM-3 (AB040767), Greenland (Z47760), Liu (AF172411), Onderstepoort (AF378705), Convac (Z35493), Snyder Hill (AF259552).

A phylogenetic tree based on the H genes of various CDV strains was generated using the MegAlign of DNAStar software. As shown in Fig. 2, the selected two samples were grouped into wild-type viruses and belonged to a genotype that is obviously different from the CDV vaccine strain.

## Discussion

To date, CDV remains one of the most important canine diseases worldwide. Surveillance represents a primary concern in the control of CDV. In addition to traditional methods using virus isolation, several promising antigen-ELISA, AGP, and FA methods have been developed and evaluated. However, these methods are laborious and time consuming. Moreover, these tests cannot differentiate CDV wild-type strains from vaccine strains[13,14].

With the recent availability of genomic sequences, molecular diagnostic methods for detection of viruses have significantly improved[15]. Complementary DNA and RNA probes have been used to detect RNA and mRNA of the CDV genome with improved specificity and sensitivity[6,7]. Primers P1 and P4 (specific for CDV and conserved among CDV species), primer P2 (specific for CDV wild-type strain), and primer P3, P5 (specific for CDV vaccine strain), were selected from the well-conserved regions of the gene encoding matrix protein. A fragment of 600 bp was consistently amplified by RT-PCR with P1/P4 for either CDV vaccine strain or wild-type strain. The nested PCR with P2/P4 generated a 247 bp fragment only for the wild-type strain, while P3/P4, P5/P6 generated a same 177 bp fragment only for the vaccine strain, and both fragments could be amplified from the mixture of CDV vaccine and wild-type strains. No amplification was obtained for NDV and other common canine viruses, such as CCV, CPV, CAV, RV, and uninfected cells control, indicating the high specificity of the method. The method was sensitive, in that it could detect as little as 0.1 TCID_50 _of the virus. The selected two samples were classified into a branch which belongs to the wild-type strain, but constituting a genotype different from that of CDV vaccine strains, as revealed by phylogenetic analysis based on the sequences of the H gene region, which is believed to be the most reliable classification and genetic typing. RT-nPCR was used to detect the 30 field samples in Heilongjiang and Jilin provinces; 20 of the field samples were CDV-positive, among which 15 were wild-type strain, and 5 were vaccine strain.

Caideron et al [16] performed an extensive phylogenetic and molecular evolution analysis on complete sequences of all CDV genes to assess the role of selection and recombination in shaping viral genetic diversity and driving the emergence of CDV in non-dog hosts. They tested the specific hypothesis that molecular adaptation at known receptor-binding sites of the haemagglutinin gene is associated with independent instances of the spread of CDV to novel non-dog hosts in the wild. The selected two samples isolated from dogs were classified into a branch which belongs to the wild-type strain, but constituting a genotype different from that of CDV vaccine strains, as revealed by phylogenetic analysis based on the sequences of the H gene region, which is believed to be the most reliable classification and genetic typing. RT-nPCR was used to detect the 51 field samples in Shandong provinces; 36 of the field samples were CDV-positive, among which 20 were wild-type strain, 16 were vaccine strain, and 4 were co-infected by wild-type and vaccine strains. In summary, the multiplex RT-nPCR developed in this study is a highly specific and sensitive assay for the rapid detection and differentiation of wild-type and vaccine strains of CDV.

For lack of other vaccine strains except CDV Onderstepoort strain, the primers P5 and P6 were designed, but could not use in this time, however, much work needs to be done before a conclusion that this method has an outstanding performance with other CDV vaccine strains. In summary, the multiplex RT-nPCR developed in this study is a highly specific and sensitive assay for the rapid detection and differentiation of wild-type and vaccine strains of CDV.

## Competing interests

The authors declare that they have no competing interests.

## Authors' contributions

WS, SZ, ZW carried out the experiments and wrote the manuscript. SC conceived the studies and participated in experimental design and coordination. All authors read and approved the final manuscript.
